# Genomic evaluation of late-term abortion in cows recorded through Dairy Herd Improvement test plans

**DOI:** 10.3168/jdsc.2022-0341

**Published:** 2023-07-13

**Authors:** M. Neupane, J.L. Hutchison, J.B. Cole, C.P. Van Tassell, P.M. VanRaden

**Affiliations:** Animal Genomics and Improvement Laboratory, Agricultural Research Service, USDA, Beltsville, MD 20705-2350

## Abstract

Late-term abortions cause significant economic loss and are of great concern for dairy herds. Late-term abortions >152 d and <251 d of gestation that terminate a lactation or initiate a new lactation have long been recorded by Dairy Herd Improvement (DHI). For 24.8 million DHI lactations, the average recorded incidence of late-term abortions across all years (2001–2018) was 1.2%. However, the 1.3% incidence of abortions reported in 2012 has declined to <1.0% incidence since 2015. Small adjustments were applied to the 82 million daughter pregnancy rate (DPR), 29 million cow conception rate (CCR), and 9 million heifer conception rate (HCR) records to account for late-term abortions more accurately. Fertility credits for CCR and HCR were changed to treat the last breeding as a failure instead of success if the next calving was coded as a late-term abortion. Similarly, when computing DPR, days open is now set to the maximum value of 250 instead of the reported days open if the next reported calving is an abortion. The test of these changes showed very small changes in standard deviation and high correlations (0.997) of adjusted predicted transmitting abilities (PTA) with official PTA from about 20,000 Holstein bulls born since 2000 with >50% reliability. For late-term abortion as a trait, estimated heritability was only 0.001 and PTA had a standard deviation of only 0.1% for recent sires with high reliability (>75%). Young animal genomic PTA have near 50% reliability but range only from −0.5 to +0.4 because of the low incidence and heritability. Genetic trend was slightly favorable and late-term abortion PTA were correlated favorably by 0.27 with net merit, 0.49 with productive life, 0.33 with livability, 0.23 with CCR, 0.20 with HCR, 0.26 with DPR, −0.31 with somatic cell score, −0.24 with daughter stillbirth, and −0.26 with daughter dystocia. Thus, PTA for late-term abortions should not be needed as a separate fertility trait and instead these minor edit changes should suffice. The PTA for late-term abortions would add little value because national evaluations for current fertility traits already account for those economic losses.

## Highlights

• Prevalence of late-term abortion is decreasing in DHI herds.
• PTA for late-term abortions would add little value because national evaluations for current fertility traits already account for those economic losses.
• Herd owners desiring more reduction in late-term abortion incidence should emphasize productive life, cow conception rate, daughter pregnancy rate, and daughter stillbirth.

## Graphical Abstract

**Figure:**
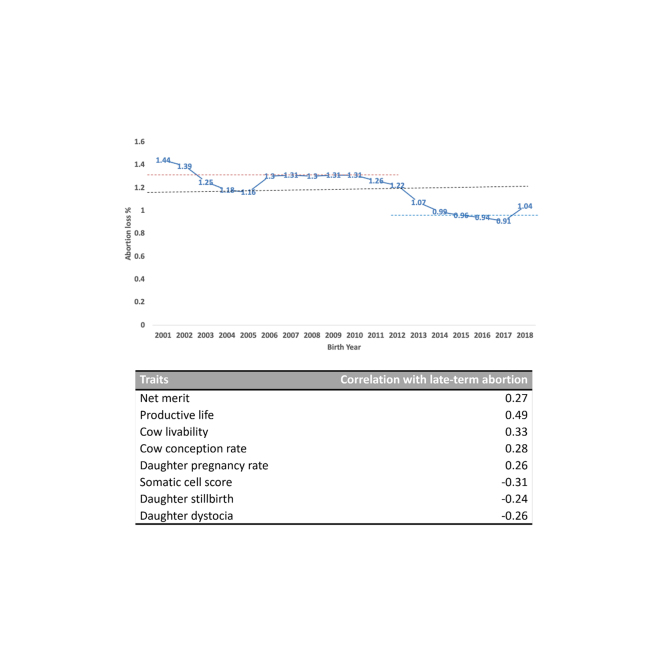
**Summary:** Late-term abortions >152 days and <251 days of gestation that terminate a lactation or initiate a new lactation have long been recorded by Dairy Herd Improvement (DHI). For 22.7 million DHI lactations, the average recorded incidence of late-term abortions across all years (2001–2018) was 1.2%. However, the 1.3% incidence of late-term abortions reported in 2012 has declined to <1.0% incidence since 2015. For late-term abortion as a trait, estimated heritability was only 0.001 and predicted transmitting abilities (PTA) had a standard deviation of only 0.1% for recent sires with high reliability (>75%). Thus, PTA for late-term abortions should not be used as a separate fertility trait because national evaluations for current fertility traits already account for those economic losses.

Abortions occurring in late gestation cause significant economic loss and are of great concern for dairy herds. The estimated cost of an abortion to the producer ranges from $90 to $1,900 and depends upon the gestation stage, labor, replacement, and veterinary costs (De Vries, 2006). The causes of abortion include infectious agents (bacteria, viruses, protozoa, and fungi), toxic agents, heat stress, genetic abnormalities, and unknown causes (Hovingh, 2009). Several research projects have shown a genetic component of abortion in cattle. Various recessive alleles and haplotypes have been associated with fetal or embryonic loss in cattle (VanRaden et al., 2011b; Cole et al., 2016). Many loci, genes, and pathways are associated with different stages of pregnancy loss in dairy cows (Gershoni et al., 2020; Sigdel et al., 2021). Even with the recent advancement in reproductive management strategies along with use of genomic selection, abortion loss continues to be a serious issue on dairy farms. The objectives of this study were to estimate the current prevalence of late-term abortions in US dairy herds, develop genomic and genomic evaluation of late-term abortion loss, and estimate correlations between the PTA for late-term abortion and other traits already included in the US dairy genetic evaluation system. This study was also used to determine whether a need exists for a separate genomic evaluation for late-term abortion or minor changes in current reproductive traits are sufficient to include late-term abortion loss.

In this study, late-term abortion was examined from 12,185,817 cows with a total of 24.8 million DHI lactation records between years 2001 to 2018 from the national cooperator database maintained by the Council on Dairy Cattle Breeding (**CDCB**; Bowie, MD). Abortions from heifers were not included in this study. Abortions were defined as those >152 d and <251 d of gestation that terminate a lactation or initiate a new lactation. The binary phenotype used for analysis was 0 (no abortion) or 100 (abortion). The average recorded incidence of late-term abortions across all years (2001–2018) was 1.2%. However, the 1.3% incidence of abortions reported in 2012 (Norman et al., 2012) has declined to <1.0% incidence since 2015 (Figure 1). This recent decrease in late-term abortions might be due to better management in DHI herds as compared with other herds, more emphasis on fertility traits, and improvements in management practices. Although there are no national surveys on abortion frequency in the United States, Sigdel et al. (2022) estimated 4.7% to 14.1% fetal loss (>42 d of gestation), whereas Hovingh (2009) estimated 3 to 5% of similar loss in US Holsteins. Similarly, 1.5% of abortion frequency was estimated in 507 Danish herds (Carpenter et al., 2006). The higher incidence in those studies might be the result of including early-term abortions with late-term abortions. Moreover, many late-term abortion losses go undetected or unreported. The lower abortion frequency in this study may result from under-reporting of losses (Parker Gaddis et al., 2012) or better management practices in DHI herds. Seasonal differences in late-term abortion loss across all the years are presented in Figure 2. The frequency of abortion is decreased in November through May and increased in June through October. Carpenter et al. (2006) also reported higher abortion loss in summer months with a peak in July. The exact cause of this seasonal difference is unknown but could be the effects of temperature and humidity on cow health or the spread of infectious agents (Norman et al., 2012).

**Figure 1:**
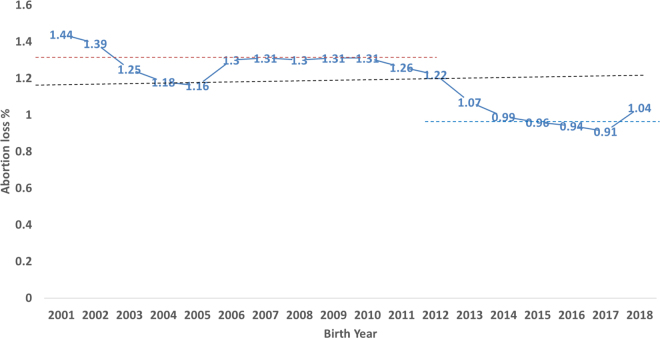
Late-term abortion loss for animals in DHI herds born from years 2001 to 2018. The black, red, and blue dotted lines indicate the average late-term abortion loss across all years (1.18%), abortions reported from years 2001 to 2012 (1.3%), and abortion reported from years 2012 to 2018 (0.99%), respectively.

**Figure 2:**
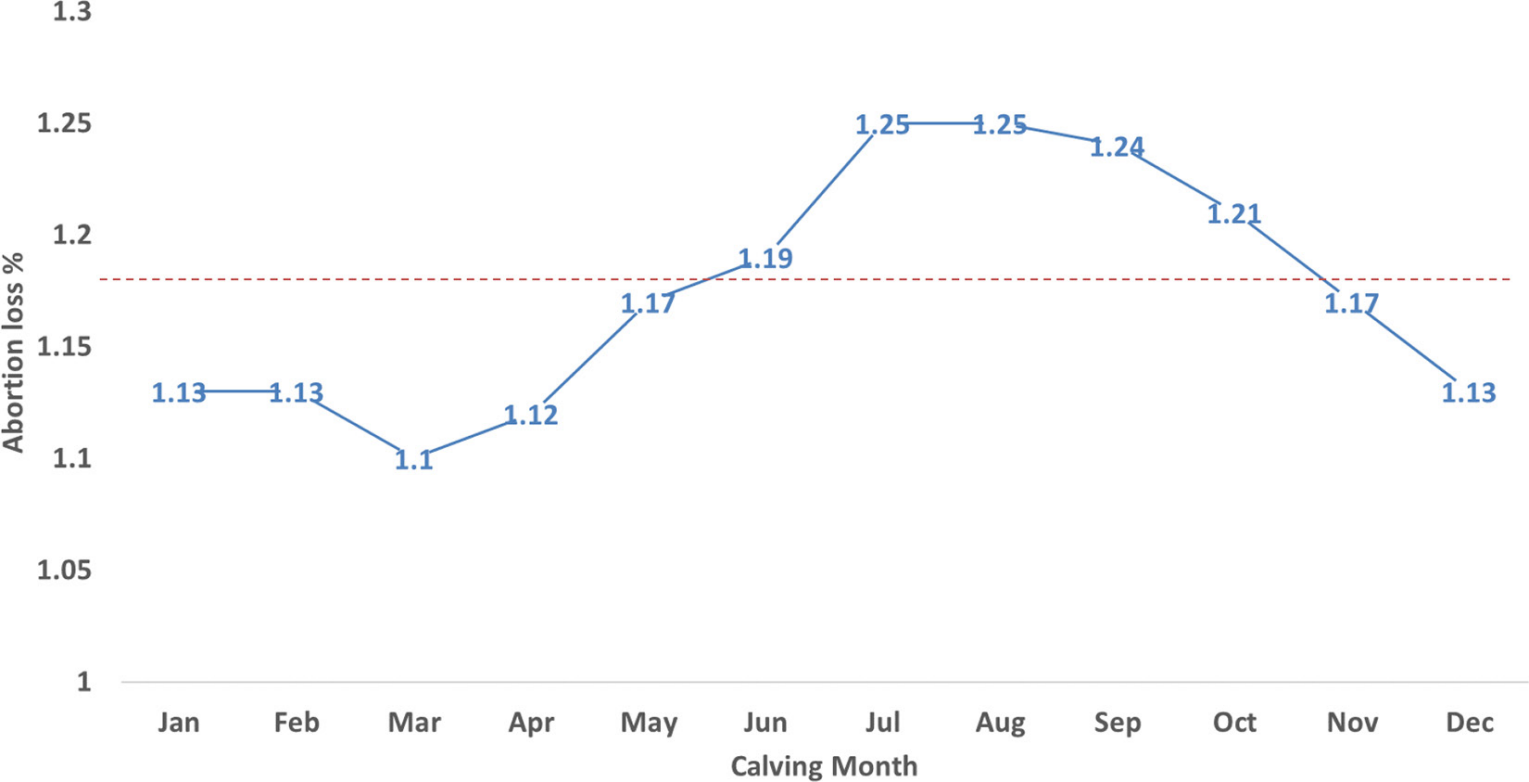
Monthly distribution of late-term abortion loss for animals in DHI herds born from years 2001 to 2018. The dotted red line indicates the average late-term abortion loss across all years (1.18%).

Predicted transmitting abilities for late-term abortion were calculated using the following animal model:

late-term abortion = HYS + PG + a + pe + e,

where late-term abortion is binary trait 0 (no abortion) or 100 (abortion), HYS is the fixed effect of herd-year-season of birth, PG is the fixed effect of parity group, a is the random additive genetic effect, pe is the permanent environmental effect, and e is the residual error. Animal, permanent environmental, and residual effects were distributed as $N\left(0,A\sigma_{a}^{2}\right),$ $N\left(0,I\sigma_{pe}^{2}\right),$ and $N\left(0,I\sigma_{e}^{2}\right),$ respectively, where **A** is the numerator relationship matrix, **I** is an identity matrix, $\sigma_{a}^{2}$ is the additive genetic variance, $\sigma_{pe}^{2}$ is the permanent environmental variance, and $\sigma_{e}^{2}$ is the residual variance. Traditional (pedigree-based) PTA were estimated using a model similar to that used for routine national genetic evaluations (VanRaden et al., 2014). Heritability was estimated using sire model REML (VanRaden, 1986). The REML estimation was performed using 4,244,756 first lactations or 59% of the total in 566,108 herd-year-seasons with seasons defined as 3-mo groups. The full animal model with 24.8 million phenotypes and 85 million animals in the pedigree used the same software as other nationally evaluated traits (VanRaden et al., 2014) with reliability estimates based on methods of VanRaden and Wiggans (1991). The model converged in 20 iterations. Variance components (REML estimates ± SE) were 0.026 ± 0.003 for sire variance and 102.20 ± 0.11 for error variance, resulting in an estimated heritability of 0.0010 ± 0.0001. Predicted transmitting abilities and genomic PTA were calculated only for Holstein. For genomic PTA calculation, 2.9 million Holsteins with genotypes were used. Animals genotyped on various platforms were imputed to 79,294 markers using Findhap version 3 (VanRaden et al., 2011a) and used in genomic evaluation.

To more accurately account for abortion losses in fertility evaluations, small adjustments were applied to trait definitions for the 82 million daughter pregnancy rate (**DPR**), 29 million cow conception rate (**CCR**), and 9 million heifer conception rate (**HCR**) records. Fertility credits for CCR and HCR were changed to treat the last breeding as a failure instead of success if the next calving was coded as a late-term abortion. Similarly, when computing DPR, days open is now set to a maximum value of 250 d instead of the reported days open if the next reported calving is an abortion. These revisions changed the DPR, CCR, and HCR definitions slightly to include more late-term pregnancy losses but affect only about 1% of lactations. The test of these changes showed very small changes in standard deviation and high correlations (0.997) of adjusted PTA with official PTA from about 20,000 Holstein bulls born since 2000 with >50% reliability. These changes accounted for some additional value of late-term abortions, but the genetic variance is very small. Because yield trait PTA exclude records initiated by an abortion, a bull's true merit for yield may be a little less than published PTA if more daughters abort.

If one considers late-term abortion as a trait, heritability was estimated at only 0.001 and the standard deviation of PTA was only 0.1% for recent sires with high reliability (>75%). Reliability of genomic PTA for young animals near 50%, but only ranged from −0.5 to +0.4 because of the low incidence and small heritability. This low level of heritability was also reported in other health and reproductive traits (Parker Gaddis et al., 2014; Sigdel et al., 2021). Correlations between PTA late-term abortion and other important traits included in US genetic evaluation system are presented in Table 1. Trait correlations were estimated using 399 bulls with >50% reliability except for calving traits (daughter stillbirth, **DSB**, and daughter dystocia) which used 62 bulls with >75% reliability. Genetic trend has increased slightly, and late-term abortion PTA were correlated favorably by 0.27 with net merit, 0.49 with productive life (**PL**), 0.33 with livability, 0.23 with CCR, 0.20 with HCR, 0.26 with DPR, −0.31 with SCS, −0.24 with DSB, and −0.26 with daughter dystocia. In this study, daughter dystocia was used to define percentage of difficult birth for heifers instead of commonly used daughter calving ease. However, in this study late-term abortion correlations were not significant with milk, fat, protein, and gestational length.

**Table 1:** Correlations (Pearson product-moment) among sire evaluations for late-term abortion and other traits included in the US dairy cattle genetic evaluation system^1^

Trait	Correlation
Net merit	0.27**
Milk	−0.04
Fat	−0.02
Protein	−0.06
Productive life	0.49**
Cow livability	0.33**
Daughter pregnancy rate	0.26**
Cow conception rate	0.23**
Heifer conception rate	0.20**
Somatic cell score	−0.31**
Gestation length	0.01
Daughter stillbirth	−0.24*
Daughter dystocia	−0.26*

1 Trait correlation estimations were based on 399 bulls with >50% reliability except for calving traits (daughter stillbirth and daughter dystocia), which used 62 bulls with >75% reliability.

* *P* < 0.01

** *P* < 0.0001.

Zoetis developed Dairy Wellness Profit (DWP$) that includes cow abortion as a new wellness trait since 2020. This study defined abortions >41 and <261 d of gestation for 3.8 million non-DHI lactation with an incidence of 11%. At least part of the reason for the higher rate of abortions in that report is because early abortions calculated from pregnancy exams were also included (Vukasinovic et al., 2017; Wijma et al., 2022). The estimate of $90 to $900 per abortion (De Vries, 2006; Wijma et al., 2022) included increased number of inseminations (fully accounted for by CCR), culling (fully accounted for by PL), loss of pregnancy (mostly accounted for by DPR), maintenance (mostly accounted for in PL), and medical expenses. So, current national fertility traits from CDCB already account for most costs of abortions.

In summary, PTA for late-term abortions are not needed as a separate fertility trait and minor edits of fertility and PL traits should adequately adjust for most effects on reproductive traits. These changes to existing fertility traits were developed and implemented in current US genomic evaluation since April 2021 (https://uscdcb.com/genomic-evaluations/). Predicted transmitting abilities for late-term abortions would add little value because national evaluations for current fertility traits already account for those economic losses. Herd owners desiring more reduction in abortion incidence should emphasize PL, CCR, DPR, and DSB more than the economic values indicate. Genomic analysis of the national abortion data might help scientists and producers better understand this trait and its correlations and influence on other published traits. However, a new late-term abortion trait might also confuse the public and add little value to the US dairy genetic evaluation system.
